# Tris(2,2′-bipyridyl-κ^2^ *N*,*N*′)copper(II) sulfate 7.5-hydrate

**DOI:** 10.1107/S1600536808043821

**Published:** 2009-01-08

**Authors:** Feng Xu, Wei You, Wei Huang

**Affiliations:** a State Key Laboratory of Coordination Chemistry, Nanjing National Laboratory of Microstructures, School of Chemistry and Chemical Engineering, Nanjing University, Nanjing, 210093, People’s Republic of China

## Abstract

The title compound, [Cu(C_10_H_8_N_2_)_3_]SO_4_·7.5H_2_O, is a six-coordinate copper(II) complex with a slightly elongated octa­hedral coordination geometry. The pyridyl rings of the three bipyridyl ligands are not coplanar, making dihedral angels of 9.5 (5), 5.2 (4) and 5.8 (5)°. In the crystal, several O—H⋯O and C—H⋯O hydrogen-bonding inter­actions are observed due to the existance of a large number of water mol­ecules and the sulfate dianions.

## Related literature

For related compounds, see Anderson (1972 ▶); Wada *et al.* (1976 ▶); Liu *et al.* (1991 ▶); Majumdar *et al.* (1998 ▶); Pavlishchuk *et al.* (1999 ▶); Murphy *et al.* (2006 ▶); Huang (2007 ▶); Wang *et al.* (2007 ▶).
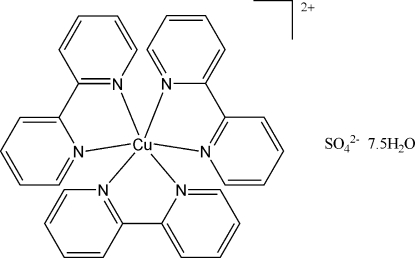


## Experimental

### 

#### Crystal data

[Cu(C_10_H_8_N_2_)_3_]SO_4_·7.5H_2_O
*M* *_r_* = 763.87
Monoclinic, 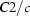

*a* = 22.857 (5) Å
*b* = 13.550 (3) Å
*c* = 24.709 (5) Å
β = 114.753 (3)°
*V* = 6950 (2) Å^3^
*Z* = 8
Mo *K*α radiation
μ = 0.76 mm^−1^
*T* = 291 (2) K
0.16 × 0.14 × 0.12 mm

#### Data collection

Bruker SMART APEX CCD area-detector diffractometer
Absorption correction: multi-scan (*SADABS*; Bruker, 2000 ▶) *T*_min_ = 0.889, *T*_max_ = 0.915
17073 measured reflections
6101 independent reflections
3819 reflections with *I* > 2σ(*I*)
*R*_int_ = 0.118

#### Refinement

*R*[*F* ^2^ > 2σ(*F* ^2^)] = 0.073
*wR*(*F* ^2^) = 0.209
*S* = 1.02
6101 reflections
447 parameters
1 restraint
H-atom parameters constrained
Δρ_max_ = 1.05 e Å^−3^
Δρ_min_ = −0.66 e Å^−3^

### 

Data collection: *SMART* (Bruker 2000 ▶); cell refinement: *SAINT* (Bruker 2000 ▶); data reduction: *SAINT*; program(s) used to solve structure: *SHELXTL* (Sheldrick, 2008 ▶); program(s) used to refine structure: *SHELXTL*; molecular graphics: *SHELXTL*; software used to prepare material for publication: *SHELXTL*.

## Supplementary Material

Crystal structure: contains datablocks global, I. DOI: 10.1107/S1600536808043821/at2687sup1.cif

Structure factors: contains datablocks I. DOI: 10.1107/S1600536808043821/at2687Isup2.hkl

Additional supplementary materials:  crystallographic information; 3D view; checkCIF report

## Figures and Tables

**Table 1:** Selected geometric parameters (Å, °)

Cu1—N5	2.061 (4)
Cu1—N2	2.076 (4)
Cu1—N1	2.092 (4)
Cu1—N4	2.100 (4)
Cu1—N3	2.173 (4)
Cu1—N6	2.176 (4)

**Table 2:** Hydrogen-bond geometry (Å, °)

*D*—H⋯*A*	*D*—H	H⋯*A*	*D*⋯*A*	*D*—H⋯*A*
O6—H6*C*⋯O1	0.85	1.98	2.570 (9)	125
O6—H6*D*⋯O1^i^	0.85	2.09	2.570 (9)	115
O7—H7*A*⋯O7^ii^	0.85	2.46	2.89 (3)	112
O7—H7*B*⋯O3	0.96	2.04	2.840 (14)	139
O8—H8*A*⋯O12	0.89	2.21	2.857 (12)	129
O9—H9*A*⋯O10	0.85	2.20	3.001 (13)	157
O10—H10*B*⋯O11	0.85	2.23	2.901 (9)	136
O12—H12*A*⋯O8	0.83	2.54	2.857 (12)	104
C17—H17⋯O6^iii^	0.93	2.56	3.489 (11)	175
C18—H18⋯O2^iv^	0.93	2.41	3.174 (12)	139
C28—H28⋯O4^i^	0.93	2.52	3.215 (9)	132

Symmetry codes: (i) 
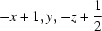
; (ii) 
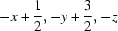
; (iii) 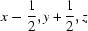; (iv) 
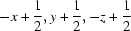
.

**Fig. 1.**
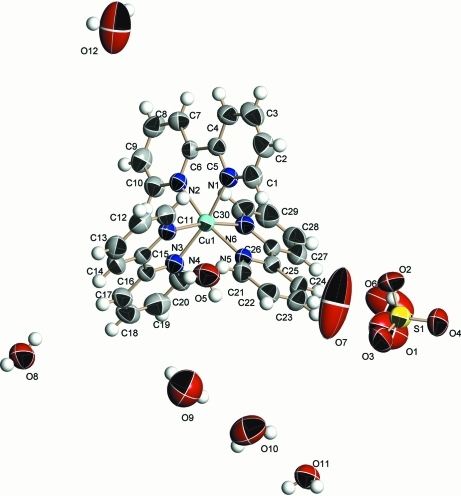
An ORTEP drawing of the title compound with the atom-numbering scheme. Displacement ellipsoids are drawn at the 50% probability level.

## supplementary crystallographic information

### Comment

The structures of tris(2,2'-bipyridyl)copper(II) hexafluoridophosphate (Wang *et al.*, 2007), tetrafluoridoborate (Huang, 2007), perchlorate (Anderson, 1972; Liu *et al.*, 1991; Majumdar *et al.*, 1998; Pavlishchuk *et al.*, 1999), and tetraphenylborate (Murphy *et al.*, 2006) have been previously reported. In addition, a similar structure of tris(2,2'-bipyridyl)nickel(II) sulfate with 7.5 water molecules (Wada *et al.*, 1976) can be found in literature. Herein, we present the crystal structure of the title compound, (I), a tris(2,2'-bipyridyl)copper(II) sulfate which is obtained as a by-product in the preparation of di(2,2'-bipyridyl)copper(II) sulfate.

The atom-numbering scheme of compound (I) is shown in Fig. 1, while selected bond distances and angles are given in Table 1. The coordination geometry of the complex about the six-coordinate Cu(II) center is slightly elongated octahedral. Four shorter Cu—N bonds occupy the equatorial plane, with a mean bond length of 2.083 (4) Å, while two axial Cu—N bonds are a little longer [Cu1—N3 = 2.173 (4) Å and Cu1—N6 = 2.176 (4) Å]. The pyridyl rings of three bipyridyl ligands are not coplanar with the dihedral angels of 9.5 (5)°, 5.2 (4)° and 5.8 (5)°, respectively. In the crystal packing of (I), quite a few O—H···O and C—H···O hydrogen bonding interactions are observed due to the existance of a large number of water molecules and the sulfate dianions.

### Experimental

The title compound was obtained from a mixed methanol/water solution of NaCl and Cu(NO_3_)_2_.3H_2_O by slow evaporation in air at room temperature.

Single crystals of compound (I) suitable for X-ray diffraction determination were formed from the filtrate as a by-product when CuSO_4_.5H_2_O (1.248 g, 5 mmol) was reacted with 2,2'-bipyridine (1.562 g, 10 mmol) in methanol (100 ml) at room temperature for 48 h. Elemental analysis: calculated for C_30_H_39_CuN_6_O_11.5_S: C 47.21, H 5.15, N 11.01%; found: C 47.11, H 5.20, N 10.89%. Main FT–IR absorptions (KBr plates, cm^-1^): 3443 (*b*), 1637 (*s*), 1606 (*m*), 1510 (*m*), 1249 (w), 1116 (*s*), 778 (*s*) and 618 (w).

### Refinement

The non-hydrogen atoms were refined anisotropically, whereas the H atoms were placed in geometrically idealized positions (C—H = 0.93 Å and O—H = 0.82–0.96 Å) and refined as riding atoms, with *U*_iso_(H) = 1.2_eq_(C) and *U*_iso_(H) = 1.5*U*_eq_(O).

### Crystal data

**Table:**

[Cu(C_10_H_8_N_2_)_3_]SO_4_·7.5H_2_O	*F*(000) = 3184
*M**_r_* = 763.27	*D*_x_ = 1.459 Mg m^−^^3^
Monoclinic, *C*2/*c*	Mo *K*α radiation, λ = 0.71073 Å
Hall symbol: -C 2yc	Cell parameters from 3884 reflections
*a* = 22.857 (5) Å	θ = 2.2–23.0°
*b* = 13.550 (3) Å	µ = 0.76 mm^−^^1^
*c* = 24.709 (5) Å	*T* = 291 K
β = 114.753 (3)°	Block, red
*V* = 6950 (2) Å^3^	0.16 × 0.14 × 0.12 mm
*Z* = 8	

### Data collection

**Table:**

Bruker SMART APEX CCD area-detector diffractometer	6101 independent reflections
Radiation source: fine-focus sealed tube	3819 reflections with *I* > 2σ(*I*)
graphite	*R*_int_ = 0.118
Detector resolution: 0 pixels mm^-1^	θ_max_ = 25.0°, θ_min_ = 2.0°
φ and ω scans	*h* = −27→26
Absorption correction: multi-scan (*SADABS*; Bruker, 2000)	*k* = −16→15
*T*_min_ = 0.889, *T*_max_ = 0.915	*l* = −20→29
17073 measured reflections	

### Refinement

**Table:**

Refinement on *F*^2^	Primary atom site location: structure-invariant direct methods
Least-squares matrix: full	Secondary atom site location: difference Fourier map
*R*[*F*^2^ > 2σ(*F*^2^)] = 0.073	Hydrogen site location: inferred from neighbouring sites
*wR*(*F*^2^) = 0.209	H-atom parameters constrained
*S* = 1.02	*w* = 1/[σ^2^(*F*_o_^2^) + (0.1136*P*)^2^] where *P* = (*F*_o_^2^ + 2*F*_c_^2^)/3
6101 reflections	(Δ/σ)_max_ < 0.001
447 parameters	Δρ_max_ = 1.05 e Å^−^^3^
1 restraint	Δρ_min_ = −0.65 e Å^−^^3^

### Special details

**Table:**

Experimental. The structure was solved by direct methods (Bruker, 2000) and successive difference Fourier syntheses.
Geometry. All e.s.d.'s (except the e.s.d. in the dihedral angle between two l.s. planes) are estimated using the full covariance matrix. The cell e.s.d.'s are taken into account individually in the estimation of e.s.d.'s in distances, angles and torsion angles; correlations between e.s.d.'s in cell parameters are only used when they are defined by crystal symmetry. An approximate (isotropic) treatment of cell e.s.d.'s is used for estimating e.s.d.'s involving l.s. planes.
Refinement. Refinement of *F*^2^ against ALL reflections. The weighted *R*-factor *wR* and goodness of fit *S* are based on *F*^2^, conventional *R*-factors *R* are based on *F*, with *F* set to zero for negative *F*^2^. The threshold expression of *F*^2^ > σ(*F*^2^) is used only for calculating *R*-factors(gt) *etc*. and is not relevant to the choice of reflections for refinement. *R*-factors based on *F*^2^ are statistically about twice as large as those based on *F*, and *R*- factors based on ALL data will be even larger.

### Fractional atomic coordinates and isotropic or equivalent isotropic displacement parameters (Å^2^)

**Table:**

	*x*	*y*	*z*	*U*_iso_*/*U*_eq_	Occ. (<1)
S1	0.42784 (10)	0.57281 (13)	0.08973 (9)	0.0894 (6)	
C1	0.1324 (3)	0.4083 (5)	0.1408 (2)	0.0708 (15)	
H1	0.1529	0.4524	0.1255	0.085*	
C2	0.1067 (3)	0.3220 (5)	0.1096 (3)	0.0824 (18)	
H2	0.1078	0.3102	0.0729	0.099*	
C3	0.0801 (3)	0.2555 (5)	0.1331 (3)	0.0874 (19)	
H3	0.0643	0.1960	0.1137	0.105*	
C4	0.0767 (3)	0.2770 (4)	0.1864 (3)	0.0684 (15)	
H4	0.0577	0.2326	0.2029	0.082*	
C5	0.1016 (2)	0.3641 (4)	0.2148 (2)	0.0498 (12)	
C6	0.0984 (2)	0.3943 (4)	0.2711 (2)	0.0498 (11)	
C7	0.0800 (3)	0.3304 (4)	0.3051 (2)	0.0636 (14)	
H7	0.0683	0.2658	0.2927	0.076*	
C8	0.0795 (3)	0.3641 (5)	0.3575 (3)	0.0728 (16)	
H8	0.0678	0.3224	0.3812	0.087*	
C9	0.0964 (3)	0.4602 (5)	0.3742 (3)	0.0756 (17)	
H9	0.0957	0.4853	0.4090	0.091*	
C10	0.1142 (3)	0.5172 (5)	0.3392 (2)	0.0678 (15)	
H10	0.1260	0.5821	0.3510	0.081*	
C11	0.0271 (3)	0.6205 (5)	0.1325 (3)	0.0721 (16)	
H11	0.0300	0.5599	0.1161	0.087*	
C12	−0.0242 (3)	0.6800 (5)	0.1014 (3)	0.0790 (17)	
H12	−0.0561	0.6601	0.0650	0.095*	
C13	−0.0273 (3)	0.7690 (5)	0.1253 (3)	0.0802 (18)	
H13	−0.0615	0.8111	0.1045	0.096*	
C14	0.0183 (3)	0.7973 (4)	0.1784 (3)	0.0705 (15)	
H14	0.0154	0.8582	0.1945	0.085*	
C15	0.0698 (3)	0.7343 (4)	0.2089 (2)	0.0582 (13)	
C16	0.1229 (3)	0.7589 (4)	0.2661 (2)	0.0560 (13)	
C17	0.1249 (3)	0.8453 (4)	0.2971 (3)	0.0715 (16)	
H17	0.0912	0.8904	0.2821	0.086*	
C18	0.1770 (4)	0.8635 (5)	0.3499 (3)	0.0830 (19)	
H18	0.1785	0.9204	0.3715	0.100*	
C19	0.2264 (4)	0.7978 (6)	0.3705 (3)	0.090 (2)	
H19	0.2625	0.8091	0.4059	0.108*	
C20	0.2214 (3)	0.7145 (5)	0.3375 (3)	0.0778 (17)	
H20	0.2553	0.6697	0.3513	0.093*	
C21	0.1931 (3)	0.6569 (4)	0.1482 (3)	0.0675 (15)	
H21	0.1500	0.6752	0.1313	0.081*	
C22	0.2310 (3)	0.6824 (4)	0.1188 (3)	0.0761 (17)	
H22	0.2140	0.7171	0.0831	0.091*	
C23	0.2949 (3)	0.6545 (5)	0.1444 (3)	0.0766 (17)	
H23	0.3216	0.6690	0.1256	0.092*	
C24	0.3184 (3)	0.6059 (4)	0.1970 (3)	0.0657 (15)	
H24	0.3618	0.5890	0.2149	0.079*	
C25	0.2786 (2)	0.5809 (4)	0.2246 (2)	0.0534 (12)	
C26	0.3003 (2)	0.5266 (4)	0.2811 (2)	0.0546 (13)	
C27	0.3634 (3)	0.5014 (4)	0.3150 (3)	0.0696 (15)	
H27	0.3949	0.5171	0.3019	0.084*	
C28	0.3800 (3)	0.4535 (4)	0.3678 (3)	0.0820 (19)	
H28	0.4228	0.4376	0.3913	0.098*	
C29	0.3331 (3)	0.4290 (5)	0.3860 (3)	0.0830 (19)	
H29	0.3434	0.3959	0.4218	0.100*	
C30	0.2710 (3)	0.4539 (4)	0.3507 (3)	0.0740 (17)	
H30	0.2392	0.4366	0.3631	0.089*	
Cu1	0.15887 (3)	0.56061 (4)	0.24096 (2)	0.0490 (3)	
N1	0.1287 (2)	0.4300 (3)	0.19183 (18)	0.0559 (10)	
N2	0.11595 (19)	0.4868 (3)	0.28857 (17)	0.0516 (10)	
N3	0.0741 (2)	0.6457 (3)	0.1861 (2)	0.0590 (11)	
N4	0.1712 (2)	0.6944 (3)	0.2874 (2)	0.0601 (11)	
N5	0.2159 (2)	0.6072 (3)	0.19974 (19)	0.0537 (10)	
N6	0.2534 (2)	0.5025 (3)	0.29848 (19)	0.0585 (11)	
O1	0.4568 (5)	0.6078 (7)	0.1493 (3)	0.200 (4)	
O2	0.3827 (4)	0.4996 (4)	0.0815 (4)	0.174 (3)	
O3	0.3951 (3)	0.6614 (4)	0.0555 (3)	0.137 (2)	
O4	0.4760 (2)	0.5401 (4)	0.0702 (2)	0.1045 (16)	
O5	0.0637 (2)	0.7425 (3)	0.0186 (2)	0.1016 (14)	
H5A	0.0291	0.7750	0.0005	0.152*	
H5B	0.0959	0.7792	0.0241	0.152*	
O6	0.5000	0.5176 (9)	0.2500	0.219 (6)	
H6D	0.5056	0.5784	0.2592	0.329*	0.50
H6C	0.4774	0.5074	0.2131	0.329*	0.50
O7	0.2595 (5)	0.6476 (14)	−0.0104 (4)	0.351 (10)	
H7A	0.2253	0.6749	−0.0123	0.526*	
H7B	0.2971	0.6645	0.0247	0.526*	
O8	0.4481 (3)	0.9589 (4)	1.0027 (3)	0.1213 (18)	
H8A	0.4197	1.0051	0.9820	0.182*	
H8B	0.4479	0.9017	0.9914	0.182*	
O9	0.6920 (4)	0.8321 (7)	0.9638 (4)	0.205 (3)	
H9A	0.7328	0.8264	0.9786	0.308*	
H9B	0.6721	0.8079	0.9831	0.308*	
O10	0.8312 (3)	0.8473 (5)	0.9860 (3)	0.164 (3)	
H10C	0.8432	0.8932	1.0118	0.245*	
H10B	0.8650	0.8238	0.9845	0.245*	
O11	0.9604 (2)	0.8820 (3)	0.9955 (2)	0.0963 (14)	
H11D	0.9513	0.9296	1.0131	0.144*	
H11C	0.9627	0.9007	0.9637	0.144*	
O12	0.3201 (4)	1.0277 (8)	0.9727 (3)	0.223 (4)	
H12A	0.3281	0.9676	0.9763	0.335*	
H12B	0.2866	1.0456	0.9762	0.335*	

### Atomic displacement parameters (Å^2^)

**Table:**

	*U*^11^	*U*^22^	*U*^33^	*U*^12^	*U*^13^	*U*^23^
S1	0.1284 (15)	0.0786 (12)	0.0998 (14)	−0.0109 (11)	0.0858 (13)	−0.0126 (10)
C1	0.083 (4)	0.079 (4)	0.061 (4)	0.009 (3)	0.040 (3)	0.001 (3)
C2	0.106 (5)	0.086 (5)	0.064 (4)	0.003 (4)	0.044 (4)	−0.016 (4)
C3	0.099 (5)	0.078 (5)	0.078 (4)	−0.001 (4)	0.029 (4)	−0.024 (4)
C4	0.075 (4)	0.060 (4)	0.071 (4)	−0.006 (3)	0.031 (3)	−0.010 (3)
C5	0.049 (3)	0.047 (3)	0.049 (3)	0.004 (2)	0.016 (2)	0.003 (2)
C6	0.050 (3)	0.050 (3)	0.054 (3)	0.006 (2)	0.026 (2)	0.004 (2)
C7	0.071 (3)	0.055 (3)	0.069 (4)	0.000 (3)	0.034 (3)	0.008 (3)
C8	0.085 (4)	0.083 (4)	0.066 (4)	−0.004 (3)	0.046 (3)	0.014 (3)
C9	0.084 (4)	0.098 (5)	0.056 (3)	0.000 (4)	0.041 (3)	−0.001 (3)
C10	0.088 (4)	0.063 (4)	0.066 (4)	0.000 (3)	0.045 (3)	−0.003 (3)
C11	0.087 (4)	0.069 (4)	0.076 (4)	−0.010 (3)	0.049 (4)	−0.016 (3)
C12	0.071 (4)	0.091 (5)	0.077 (4)	−0.010 (4)	0.034 (3)	−0.001 (4)
C13	0.081 (4)	0.081 (5)	0.087 (5)	0.014 (4)	0.044 (4)	0.019 (4)
C14	0.084 (4)	0.053 (3)	0.089 (4)	0.010 (3)	0.051 (4)	0.003 (3)
C15	0.073 (3)	0.049 (3)	0.076 (4)	−0.004 (3)	0.054 (3)	0.005 (3)
C16	0.074 (3)	0.044 (3)	0.068 (3)	−0.005 (3)	0.048 (3)	0.000 (3)
C17	0.095 (4)	0.059 (4)	0.079 (4)	−0.001 (3)	0.053 (4)	−0.003 (3)
C18	0.124 (6)	0.063 (4)	0.080 (5)	−0.013 (4)	0.061 (5)	−0.012 (3)
C19	0.105 (5)	0.093 (5)	0.075 (4)	−0.023 (4)	0.041 (4)	−0.010 (4)
C20	0.094 (5)	0.066 (4)	0.083 (5)	0.005 (3)	0.047 (4)	−0.002 (3)
C21	0.070 (3)	0.064 (4)	0.078 (4)	0.007 (3)	0.041 (3)	0.005 (3)
C22	0.106 (5)	0.067 (4)	0.076 (4)	−0.005 (4)	0.059 (4)	0.003 (3)
C23	0.088 (4)	0.080 (4)	0.087 (4)	−0.012 (4)	0.062 (4)	−0.004 (4)
C24	0.058 (3)	0.072 (4)	0.080 (4)	−0.006 (3)	0.042 (3)	−0.009 (3)
C25	0.057 (3)	0.051 (3)	0.060 (3)	−0.008 (2)	0.033 (3)	−0.012 (2)
C26	0.054 (3)	0.047 (3)	0.070 (3)	−0.005 (2)	0.033 (3)	−0.013 (3)
C27	0.059 (3)	0.067 (4)	0.083 (4)	−0.001 (3)	0.030 (3)	−0.005 (3)
C28	0.070 (4)	0.075 (4)	0.085 (5)	0.011 (3)	0.017 (4)	0.007 (4)
C29	0.086 (5)	0.079 (5)	0.078 (4)	0.014 (4)	0.029 (4)	0.018 (3)
C30	0.082 (4)	0.074 (4)	0.072 (4)	−0.005 (3)	0.039 (3)	0.012 (3)
Cu1	0.0589 (4)	0.0479 (4)	0.0492 (4)	−0.0018 (3)	0.0316 (3)	−0.0012 (3)
N1	0.066 (3)	0.057 (3)	0.050 (2)	0.001 (2)	0.030 (2)	−0.001 (2)
N2	0.060 (2)	0.055 (3)	0.047 (2)	0.001 (2)	0.0291 (19)	−0.001 (2)
N3	0.074 (3)	0.051 (3)	0.069 (3)	−0.003 (2)	0.048 (3)	−0.005 (2)
N4	0.071 (3)	0.058 (3)	0.062 (3)	0.001 (2)	0.038 (2)	−0.001 (2)
N5	0.062 (3)	0.048 (2)	0.063 (3)	0.002 (2)	0.038 (2)	0.000 (2)
N6	0.066 (3)	0.051 (3)	0.065 (3)	0.000 (2)	0.033 (2)	0.003 (2)
O1	0.327 (12)	0.198 (7)	0.112 (5)	−0.018 (7)	0.128 (7)	−0.025 (5)
O2	0.206 (7)	0.086 (4)	0.325 (10)	−0.045 (4)	0.205 (7)	−0.025 (5)
O3	0.185 (6)	0.076 (3)	0.210 (7)	0.022 (4)	0.143 (5)	0.018 (4)
O4	0.112 (4)	0.112 (4)	0.118 (4)	0.010 (3)	0.075 (3)	−0.006 (3)
O5	0.101 (3)	0.086 (3)	0.110 (3)	−0.002 (3)	0.036 (3)	0.008 (3)
O6	0.277 (14)	0.159 (10)	0.116 (7)	0.000	−0.021 (8)	0.000
O7	0.205 (9)	0.75 (3)	0.124 (6)	0.178 (14)	0.096 (6)	0.082 (12)
O8	0.155 (5)	0.098 (4)	0.145 (5)	−0.003 (3)	0.096 (4)	−0.009 (3)
O9	0.188 (7)	0.220 (9)	0.214 (8)	0.002 (7)	0.090 (7)	0.004 (7)
O10	0.123 (5)	0.180 (6)	0.164 (6)	−0.035 (5)	0.037 (4)	0.006 (5)
O11	0.131 (4)	0.085 (3)	0.090 (3)	−0.003 (3)	0.063 (3)	−0.006 (3)
O12	0.201 (8)	0.351 (12)	0.126 (5)	0.137 (8)	0.077 (5)	0.104 (7)

### Geometric parameters (Å, °)

**Table:**

S1—O2	1.384 (6)	C20—H20	0.9300
S1—O1	1.418 (7)	C21—N5	1.338 (7)
S1—O4	1.444 (5)	C21—C22	1.387 (7)
S1—O3	1.479 (6)	C21—H21	0.9300
C1—N1	1.332 (7)	C22—C23	1.377 (9)
C1—C2	1.389 (8)	C22—H22	0.9300
C1—H1	0.9300	C23—C24	1.351 (8)
C2—C3	1.346 (9)	C23—H23	0.9300
C2—H2	0.9300	C24—C25	1.388 (7)
C3—C4	1.384 (8)	C24—H24	0.9300
C3—H3	0.9300	C25—N5	1.350 (6)
C4—C5	1.369 (7)	C25—C26	1.468 (8)
C4—H4	0.9300	C26—N6	1.353 (6)
C5—N1	1.341 (6)	C26—C27	1.374 (7)
C5—C6	1.481 (7)	C27—C28	1.361 (8)
C6—N2	1.332 (6)	C27—H27	0.9300
C6—C7	1.388 (7)	C28—C29	1.364 (9)
C7—C8	1.378 (8)	C28—H28	0.9300
C7—H7	0.9300	C29—C30	1.361 (9)
C8—C9	1.372 (8)	C29—H29	0.9300
C8—H8	0.9300	C30—N6	1.351 (7)
C9—C10	1.343 (8)	C30—H30	0.9300
C9—H9	0.9300	Cu1—N5	2.061 (4)
C10—N2	1.334 (6)	Cu1—N2	2.076 (4)
C10—H10	0.9300	Cu1—N1	2.092 (4)
C11—N3	1.354 (7)	Cu1—N4	2.100 (4)
C11—C12	1.364 (8)	Cu1—N3	2.173 (4)
C11—H11	0.9300	Cu1—N6	2.176 (4)
C12—C13	1.357 (9)	O5—H5A	0.8500
C12—H12	0.9300	O5—H5B	0.8500
C13—C14	1.345 (8)	O6—H6D	0.8500
C13—H13	0.9300	O6—H6C	0.8500
C14—C15	1.393 (7)	O7—H7A	0.8497
C14—H14	0.9300	O7—H7B	0.9603
C15—N3	1.347 (6)	O8—H8A	0.8927
C15—C16	1.466 (7)	O8—H8B	0.8235
C16—N4	1.332 (6)	O9—H9A	0.8500
C16—C17	1.389 (7)	O9—H9B	0.8500
C17—C18	1.371 (9)	O10—H10C	0.8497
C17—H17	0.9300	O10—H10B	0.8504
C18—C19	1.358 (9)	O11—H11D	0.8499
C18—H18	0.9300	O11—H11C	0.8498
C19—C20	1.369 (9)	O12—H12A	0.8306
C19—H19	0.9300	O12—H12B	0.8433
C20—N4	1.317 (7)		
			
O2—S1—O1	112.8 (5)	C23—C22—C21	117.7 (6)
O2—S1—O4	111.0 (4)	C23—C22—H22	121.2
O1—S1—O4	111.1 (5)	C21—C22—H22	121.2
O2—S1—O3	109.3 (4)	C24—C23—C22	119.7 (5)
O1—S1—O3	103.0 (5)	C24—C23—H23	120.2
O4—S1—O3	109.4 (3)	C22—C23—H23	120.2
N1—C1—C2	122.1 (6)	C23—C24—C25	120.8 (5)
N1—C1—H1	118.9	C23—C24—H24	119.6
C2—C1—H1	118.9	C25—C24—H24	119.6
C3—C2—C1	119.1 (6)	N5—C25—C24	120.1 (5)
C3—C2—H2	120.4	N5—C25—C26	115.9 (4)
C1—C2—H2	120.4	C24—C25—C26	124.0 (5)
C2—C3—C4	119.0 (6)	N6—C26—C27	121.1 (5)
C2—C3—H3	120.5	N6—C26—C25	115.2 (4)
C4—C3—H3	120.5	C27—C26—C25	123.8 (5)
C5—C4—C3	119.5 (6)	C28—C27—C26	120.2 (6)
C5—C4—H4	120.2	C28—C27—H27	119.9
C3—C4—H4	120.2	C26—C27—H27	119.9
N1—C5—C4	121.6 (5)	C27—C28—C29	119.3 (6)
N1—C5—C6	115.3 (4)	C27—C28—H28	120.4
C4—C5—C6	123.1 (5)	C29—C28—H28	120.4
N2—C6—C7	121.2 (4)	C30—C29—C28	118.8 (6)
N2—C6—C5	115.9 (4)	C30—C29—H29	120.6
C7—C6—C5	122.9 (5)	C28—C29—H29	120.6
C8—C7—C6	119.1 (5)	N6—C30—C29	123.1 (6)
C8—C7—H7	120.5	N6—C30—H30	118.4
C6—C7—H7	120.5	C29—C30—H30	118.4
C9—C8—C7	119.0 (5)	N5—Cu1—N2	166.92 (16)
C9—C8—H8	120.5	N5—Cu1—N1	95.46 (16)
C7—C8—H8	120.5	N2—Cu1—N1	78.61 (16)
C10—C9—C8	118.4 (5)	N5—Cu1—N4	92.11 (16)
C10—C9—H9	120.8	N2—Cu1—N4	95.40 (16)
C8—C9—H9	120.8	N1—Cu1—N4	169.44 (17)
N2—C10—C9	124.3 (6)	N5—Cu1—N3	96.47 (16)
N2—C10—H10	117.9	N2—Cu1—N3	95.70 (15)
C9—C10—H10	117.9	N1—Cu1—N3	95.48 (16)
N3—C11—C12	123.0 (6)	N4—Cu1—N3	76.32 (18)
N3—C11—H11	118.5	N5—Cu1—N6	76.90 (17)
C12—C11—H11	118.5	N2—Cu1—N6	91.77 (16)
C13—C12—C11	118.0 (6)	N1—Cu1—N6	93.93 (16)
C13—C12—H12	121.0	N4—Cu1—N6	94.94 (17)
C11—C12—H12	121.0	N3—Cu1—N6	168.97 (16)
C14—C13—C12	121.1 (6)	C1—N1—C5	118.5 (5)
C14—C13—H13	119.4	C1—N1—Cu1	126.9 (4)
C12—C13—H13	119.4	C5—N1—Cu1	114.6 (3)
C13—C14—C15	119.1 (6)	C6—N2—C10	118.1 (4)
C13—C14—H14	120.4	C6—N2—Cu1	114.8 (3)
C15—C14—H14	120.4	C10—N2—Cu1	126.2 (4)
N3—C15—C14	120.9 (5)	C15—N3—C11	117.8 (5)
N3—C15—C16	115.4 (5)	C15—N3—Cu1	114.5 (4)
C14—C15—C16	123.6 (5)	C11—N3—Cu1	127.7 (4)
N4—C16—C17	120.4 (5)	C20—N4—C16	119.1 (5)
N4—C16—C15	116.5 (4)	C20—N4—Cu1	123.9 (4)
C17—C16—C15	123.1 (5)	C16—N4—Cu1	116.8 (4)
C18—C17—C16	119.4 (6)	C21—N5—C25	118.8 (4)
C18—C17—H17	120.3	C21—N5—Cu1	123.4 (3)
C16—C17—H17	120.3	C25—N5—Cu1	117.7 (3)
C19—C18—C17	119.5 (6)	C30—N6—C26	117.5 (5)
C19—C18—H18	120.2	C30—N6—Cu1	128.2 (4)
C17—C18—H18	120.2	C26—N6—Cu1	114.1 (3)
C18—C19—C20	117.9 (7)	H5A—O5—H5B	109.5
C18—C19—H19	121.0	H6D—O6—H6C	113.6
C20—C19—H19	121.0	H7A—O7—H7B	113.6
N4—C20—C19	123.6 (6)	H8A—O8—H8B	124.6
N4—C20—H20	118.2	H9A—O9—H9B	118.1
C19—C20—H20	118.2	H10C—O10—H10B	107.2
N5—C21—C22	123.0 (5)	H11D—O11—H11C	111.7
N5—C21—H21	118.5	H12A—O12—H12B	116.1
C22—C21—H21	118.5		

### Hydrogen-bond geometry (Å, °)

**Table:**

*D*—H···*A*	*D*—H	H···*A*	*D*···*A*	*D*—H···*A*
O6—H6C···O1	0.85	1.98	2.570 (9)	125
O6—H6D···O1^i^	0.85	2.09	2.570 (9)	115
O7—H7A···O7^ii^	0.85	2.46	2.89 (3)	112
O7—H7B···O3	0.96	2.04	2.840 (14)	139
O8—H8A···O12	0.89	2.21	2.857 (12)	129
O9—H9A···O10	0.85	2.20	3.001 (13)	157
O10—H10B···O11	0.85	2.23	2.901 (9)	136
O12—H12A···O8	0.83	2.54	2.857 (12)	104
C17—H17···O6^iii^	0.93	2.56	3.489 (11)	175
C18—H18···O2^iv^	0.93	2.41	3.174 (12)	139
C20—H20···N6	0.93	2.61	3.210 (8)	123
C28—H28···O4^i^	0.93	2.52	3.215 (9)	132

Symmetry codes: (i) −*x*+1, *y*, −*z*+1/2; (ii) −*x*+1/2, −*y*+3/2, −*z*; (iii) *x*−1/2, *y*+1/2, *z*; (iv) −*x*+1/2, *y*+1/2, −*z*+1/2.
